# A novel prognostic model based on ferritin and nomogram‐revised risk index could better stratify patients with extranodal natural killer/T‐cell lymphoma

**DOI:** 10.1002/cam4.5820

**Published:** 2023-03-16

**Authors:** Xindi Liu, Yuanzheng Liang, Ziyuan Shen, Liqiang Wei, Jing Yang, Yingshi Piao, Wei Sang, Pengfei Li, Liang Wang

**Affiliations:** ^1^ Department of Hematology, Beijing TongRen Hospital Capital Medical University Beijing China; ^2^ Department of Epidemiology and Biostatistics, School of Public Health Anhui Medical University Hefei China; ^3^ Department of Pathology, Beijing TongRen Hospital Capital Medical University Beijing China; ^4^ Beijing Key Laboratory of Head and Neck Molecular Diagnostic Pathology, Beijing TongRen Hospital Capital Medical University Beijing China; ^5^ Department of Hematology Affiliated Hospital of Xuzhou Medical University Xuzhou China; ^6^ Department of Oncology, Cancer Center, Integrated Hospital of Traditional Chinese Medicine Southern Medical University Guangzhou China

**Keywords:** extranodal NK/T‐cell lymphoma, ferritin, individualized treatment, nomogram‐revised risk index, prognostic model

## Abstract

**Background:**

Extranodal natural killer (NK)/T‐cell lymphoma (ENKTCL) is an aggressive lymphoma with marked heterogeneity, resulting in a distinct prognosis even in patients with the same disease stage. The nomogram‐revised risk index (NRI) has been proposed to stratify patients with ENKTCL. Numerous reports have revealed the prognostic role of serum ferritin in various cancers.

**Purpose:**

We aimed to evaluate the role of NRI in our single cohort of patients with ENKTCL treated uniformly, explore the prognostic value of ferritin, and establish a new prognostic model to better stratify patients with ENKTCL.

**Methods:**

We included 326 patients with ENKTCL with detailed data regarding clinical characteristics and survival outcomes. All patients were treated with asparaginase‐based chemotherapy with or without radiotherapy. Multiple R packages were used to analyze the prognostic factors and derive a novel prognostic model.

**Results:**

In the training cohort comprising 236 patients with ENKTCL, NRI significantly correlated with progression‐free survival (PFS) and overall survival (*p* < 0.0001). Using a ferritin level of 400 μg/L as the cutoff value, patients with high ferritin levels had significantly inferior PFS (*p* = 0.00028). Integrating the NRI score and four easily accessible clinical parameters, namely ferritin, hemoglobin, albumin, and D‐dimer, a new prognostic model was constructed, stratifying patients with ENKTCL into three risk groups. This new prognostic model was independent of disease stage and NRI and performed better than NRI. Furthermore, this model helped to stratify patients within the same NRI risk groups. Finally, the role of this novel prognostic model was validated in the external validation cohort comprising 90 patients with ENKTCL.

**Conclusions:**

Serum ferritin level could be a novel prognostic factor in patients with ENKTCL. The new prognostic model combining NRI and clinical parameters could better predict the prognosis of ENKTCL, thereby warranting further validation and potentially guiding individualized treatment in future prospective clinical trials.

## Graphical abstract

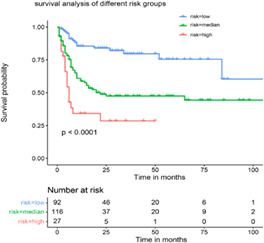

## 1 INTRODUCTION

Extranodal natural killer (NK)/T‐cell lymphoma (ENKTCL) is an aggressive malignancy associated with Epstein–Barr virus infection. In the past decade, upfront radiation therapy, immunotherapy, and non‐anthracycline‐based chemotherapy were found to largely improve the prognosis of ENKTCL.^1^, ^2^ However, considerable challenges and unmet clinical needs persist in patients with advanced and relapsed/refractory ENKTCL.

Currently, patient management involves heterogeneous strategies, primarily depending on the Ann Arbor staging system.^3^ Several prognostic models, including the International Prognostic Index (IPI),^4^ Korea Prognostic Index (KPI),^5^ and prognostic index of natural killer lymphoma (PINK),^6^ have been validated in patients with ENKTCL. However, these models have failed to predict prognosis consistently. Moreover, some of these models developed in the pre‐asparaginase era, indicating that they might lack relevance in the era of asparaginase‐based therapy. In 2020, the nomogram‐revised risk index (NRI) was reported to predict the prognosis of all patients with ENKTCL, especially early‐stage patients.^7^ Xiong et al. classified ENKTCL into TSIM, HEA, and MB subtypes according to multi‐omics analysis, and this molecular subtyping system could predict outcomes, as well as guide selection of appropriate drug therapy.^8^ Furthermore, a novel single‐nucleotide polymorphism prognostic evaluation system was established in 2022, which could be employed as an effective tool to predict the prognosis of patients with ENKTCL and determine which patient population would benefit from chemotherapy.^9^

In the present study, we examined and verified the NRI to generate additional evidence regarding its prognostic value and general applicability. Furthermore, we found that several clinical indicators, such as ferritin, might significantly impact the prognosis of patients with ENKTCL. Therefore, we constructed a new prognostic index by combining NRI and clinical indicators to further predict the prognosis of patients within the same NRI stratification.

## 2 PATIENTS AND METHODS

### 2.1 Patients and treatments

In total, 236 patients diagnosed with ENKTCL from January 2010 to December 2021 were included in our analysis as the training cohort. All patients had received initial treatment with asparaginase‐based chemotherapy with or without radiotherapy according to the stage and disease location. For patients with early‐stage disease, sandwich protocols (radiotherapy after an initial three to four cycles of asparaginase‐based regimens, followed by an additional two to three cycles of chemotherapy as consolidation) were employed. Considering patients with advanced‐stage disease, six cycles of asparaginase‐based chemotherapy were administered, with or without local radiotherapy as consolidation. The chemotherapy regimens used in this cohort were mainly GELOX/P‐GEMOX (gemcitabine, oxaliplatin, and L‐asparaginase/pegaspargase) or EPOCHL (etoposide, doxorubicin, vincristine, cyclophosphamide, prednisone, and L‐asparaginase). After three to four cycles of induction chemotherapy, patients with stage IE/IIE disease received radiotherapy, with a total dose of 50–56 Gy for the primary tumor lesion.

^18^F‐FDG positron emission tomography/computed tomography and magnetic resonance imaging (MRI) scans were performed to assess the efficacy every two cycles of chemotherapy. After completing all treatments, patients were followed up every 3 months for the first 2 years, then every 6 months for the next 3 years, and yearly thereafter, by performing clinical examination and MRI.

Moreover, we obtained an independent external cohort (*n* = 90 patients) from Xuzhou Medical University and South Medical University to validate the role of this novel prognostic model.

#### 2.1.1 Statistical analysis

The primary endpoint was progression‐free survival (PFS), defined as the time from initiating treatment to disease progression, relapse, death of any reason, or last follow‐up. Overall survival (OS) was defined as the time from initiating treatment to death attributed to any reason or last follow‐up. All data were processed by R software and respective software packages (http://www.r‐project.org/).

## 3 RESULTS

### 3.1 Clinical features and survival data of patients

**FIGURE 1:**
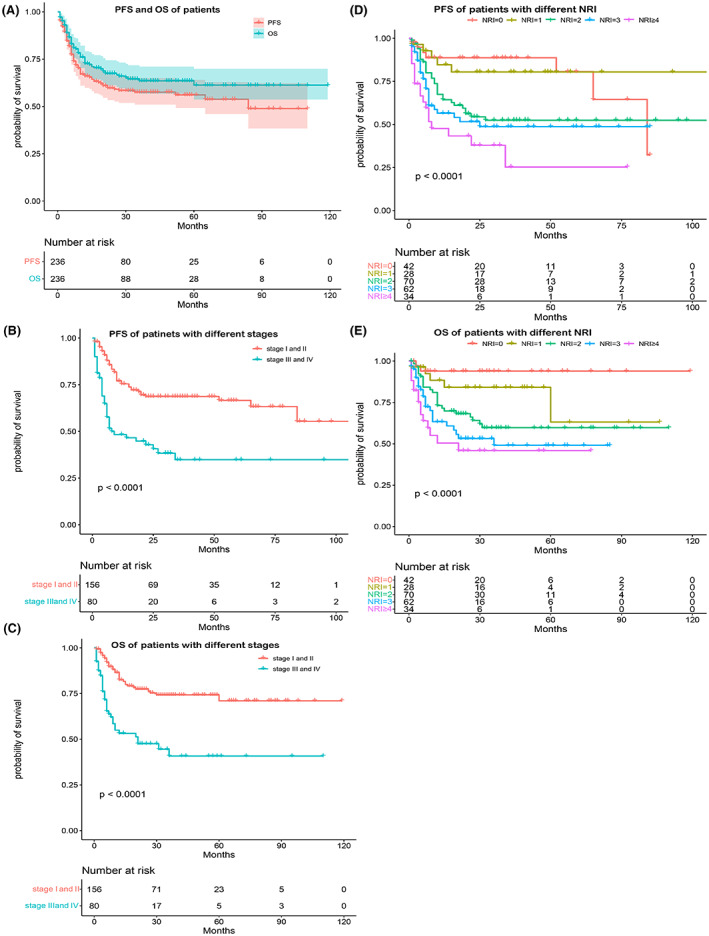
NRI and stage influence the prognosis of patients with ENKTCL. (A) The predicted 5‐year PFS and OS rates. (B, C) The predicted 5‐year PFS and OS rates with different stages (*p* < 0.0001). (D, E) The predicted 5‐year PFS and OS rates with different NRI (*p* < 0.0001). ENKTCL, extranodal natural killer/T‐cell lymphoma; NRI, nomogram‐revised risk index; OS, overall survival; PFS, progression‐free survival.

Table 1 summarizes the clinical characteristics of all 236 patients in the training cohort and 90 patients in the external validation cohort. Considering the training cohort of 236 patients, the male–female ratio was 2.37:1; the median age was 46 years. Extensive primary tumor invasion (PTI) was noted in 59.7% of patients. The majority of patients were aged ≤60 years (84.7%), with stage I/II disease (66.1%), and presented normal lactate dehydrogenase (LDH) levels (74.2%). A small number of patients exhibited lymph node involvement (33.1%). More than 50% of patients had normal levels of albumin (50.4%), hemoglobin (61.9%), and D‐dimer (D‐D) (65.3%). The percentage of patients with NRI 0‐, 1‐, 2‐, 3‐, 4‐, and 5‐points was 17.8, 11.9, 29.7, 26.3, 11.9, and 2.5%, respectively. Considering a median follow‐up time of 34 months (1–119), the predicted 5‐year PFS and OS rates were 56% and 61%, respectively (Figure 1A). Patients with early‐stage disease had significantly better 5‐year PFS and OS rates than those with advanced‐stage disease (66.6% vs. 42.9% for PFS, and 80% vs. 47.5% for OS, *p* < 0.0001) (Figure 1B,C). Patients with different NRI presented significantly different PFS and OS (*p* < 0.0001) (Figure 1D,E). As is shown in Table 1, the basic characteristics of the external validation cohort (90 patients) did not differ from those of the training cohort.

**TABLE 1:** Clinical characteristics of patients with ENKTCL.

Characteristic	Training cohort, *n* (%)	External validation cohort, *n* (%)	*p*‐value
Total number	236 (100)	90 (100)	_
Sex			0.8912
Male	166 (70.3)	64 (71.1)	
Female	70 (29.7)	26 (28.9)	
Age (years)			0.086
≤60	200 (84.7)	69 (76.7)	
>60	36 (15.3)	21 (23.3)	
Ann Arbor stage			0.3503
I	55 (23.3)	15 (16.7)	
II	101 (42.8)	45 (50.0)	
III–IV	80 (33.9)	30 (33.3)	
PTI			0.5532
No Yes	95 (40.3) 141 (59.7)	33 (36.7) 57 (63.3)	
Lymph nodes involvement			0.122
No	158 (66.9)	52 (57.8)	
Yes	78 (33.1)	38 (42.2)	
Elevated LDH			0.8803
No	175 (74.2)	66 (73.4)	
Yes	61 (25.8)	24 (26.6)	
B symptoms			0.553
No	58 (24.6)	25 (27.8)	
Yes	178 (75.4)	65 (72.2)	
Reduced albumin			0.6385
No	119 (50.4)	48 (53.3)	
Yes	117 (49.6)	42 (46.7)	
Reduced hemoglobin			0.9005
Yes	146 (61.9)	55 (61.1)	
No	90 (38.1)	35 (38.9)	
Elevated D‐dimer			0.4857
No	154 (65.3)	55 (61.1)	
Yes	82 (34.7)	35 (38.9)	
NRI (point)			0.06204
0	42 (17.8)	9 (10.0)	
1	28 (11.9)	8 (8.9)	
2	70 (29.7)	29 (32.2)	
3	62 (26.3)	37 (41.1)	
4 5	28 (11.9) 6 (2.5)	5 (5.6) 2 (2.2)	
Ferritin (μg/L)			0.2238
<400	166 (70.3)	57 (63.3)	
≥400	70 (29.7)	33 (36.7)	

Abbreviations: ENKTCL, extranodal NK/T‐cell lymphoma; LDH, lactate dehydrogenase; NRI, nomogram‐revised risk index; PTI, primary tumor infiltration.

### 3.2 Establishment of the novel NRI‐based prognostic model

**FIGURE 2:**
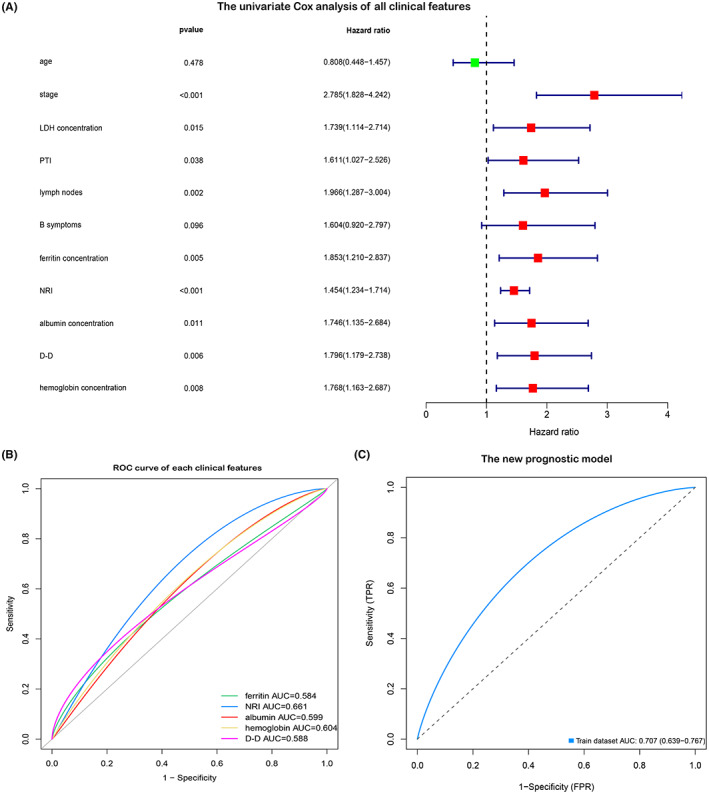
Clinical data analysis and preliminary model establishment. (A) Univariate Cox analysis of all clinical features (*p* < 0.05). (B): ROC curve of each clinical feature in the new prognostic model. (C) ROC curve of the new prognostic model. ROC, receiver operating characteristic curve.

Using univariate Cox analysis, the *p*‐value of the following factors was found to be significant (*p* < 0.05): Ann Arbor stage, LDH concentration, PTI, lymph nodes, ferritin concentration, NRI, albumin concentration, D‐D concentration, and hemoglobin concentration (Figure 2A). Given that NRI includes stage, LDH concentration, age, PTI, and other factors, and lymph node involvement and B symptoms exhibit a clear relationship with stage, we finally selected NRI, ferritin concentration, hemoglobin concentration, albumin concentration, and D‐D concentration to construct a new prognostic model. Furthermore, we used the ROC curve for further data interpretation of these clinical features. Herein, we found their AUC approximated 0.6, among which the AUC of NRI was 0.66 (Figure 2B). Considering the patient selection and regional differences, this result was more consistent with the previously reported NRI test results. However, the AUC of the new prognostic model increased to 0.707 (Figure 2C), indicating that this new model was of greater value in prognosis prediction than the NRI model. Moreover, the concentrations of ferritin, albumin, hemoglobin, and D‐D were substantially easy to obtain, reducing the limitations of this model in terms of clinical application.

### 3.3 Prognostic value of ferritin in NK/T‐cell lymphoma

**FIGURE 3:**
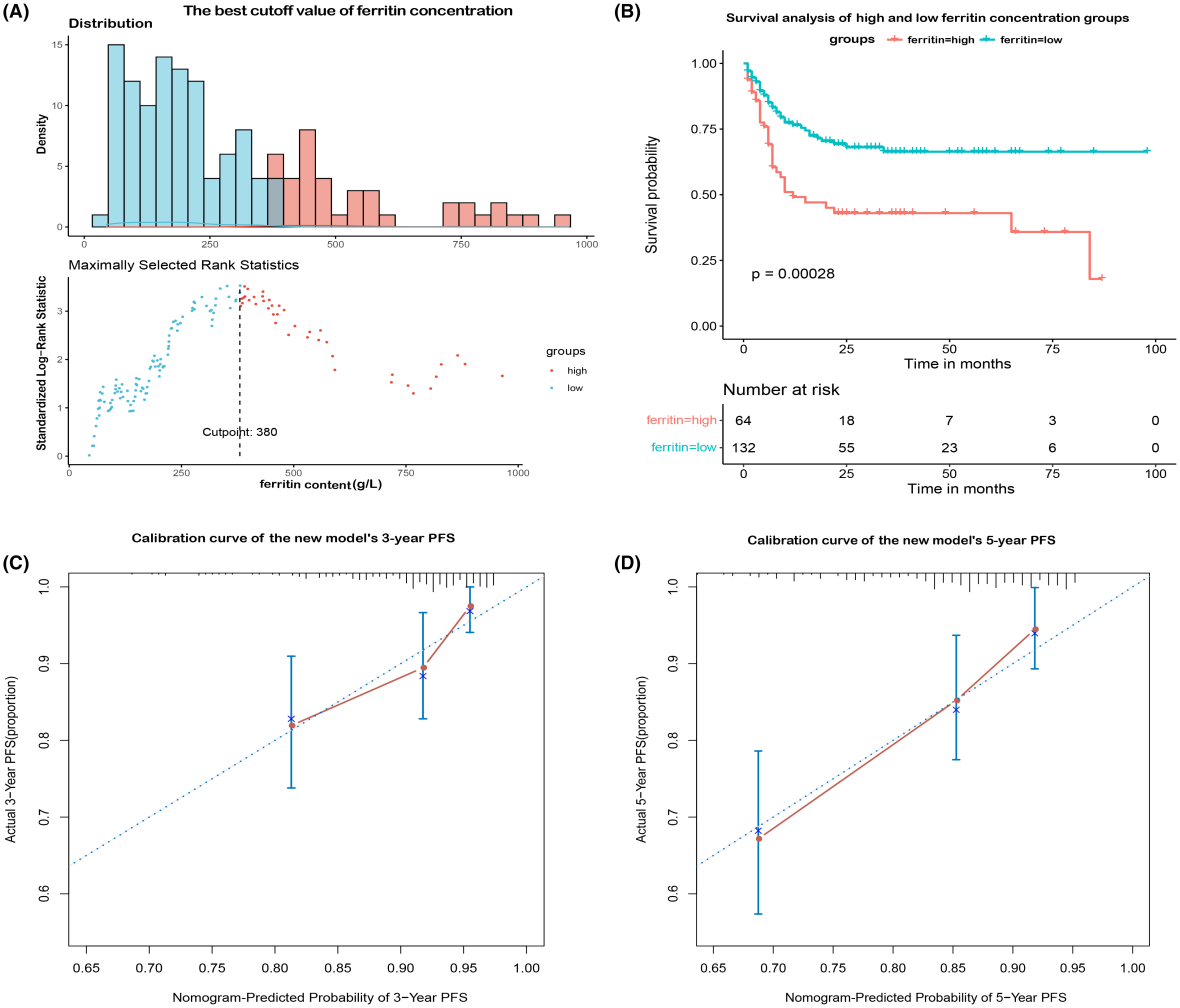
Ferritin concentration selection and a calibration curve of the model. (A) The best cutoff value of ferritin concentration (survminer R package). (B) Survival analysis of high and low ferritin concentration groups using 400 μg/L as the cutoff value (*p* = 0.00028). (C) Calibration curve of the 3‐year PFS with the new model. (D): Calibration curve of the 5‐year PFS with the new model. OS, overall survival; PFS, progression‐free survival.

We established a ferritin value of 380 μg/L as the best cutoff value in our analysis. For easy memorization and application in clinical practice, we selected 400 μg/L as the cutoff value (Figure 3A). Based on the survival analysis results, the ferritin concentration played a significant role in predicting prognosis using either 400 or 380 μg/L as the cutoff value (Figure 3B and Figure S1).

### 3.4 Construction of a novel, easily applicable NRI‐based prognostic index of ENKTCL

We used the *nomogram function* to establish the weightings of each component in the novel model described above. Among examined indicators, albumin concentration accounted for the least, whereas NRI and D‐D concentrations showed substantial accountability (Figure S2). According to the results of the univariate Cox regression analysis of the NRI score in combination with clinical data, we proposed an applicable index for clinical convenience. The final index was established using the ROC curve and nomogram. The weightings of every single component are listed in Table 2. Patients were divided into one of three risk groups by combining the sum of these parameters (low, 0–2; medium, 3–5; high, ≥6). To further evaluate the prediction role of this model, we plotted the calibration curve using 3‐ and 5‐year PFS data, respectively. (Figure 3C,D).

**TABLE 2:** The adopted weights of single new index components.

	0 point	1 point	2 points	3 points
NRI (point)	0	1–2	3–4	5
Ferritin (μg/L)	<400		≥400	
D‐dimer (mg/L)	0–0.55	0.55–1.5	1.5–5	≥5
Hemoglobin (g/L)	0–120	60–120	≤60	
Albumin (g/L)	≥35	<35		

NRI, nomogram‐revised risk index.

**FIGURE 4:**
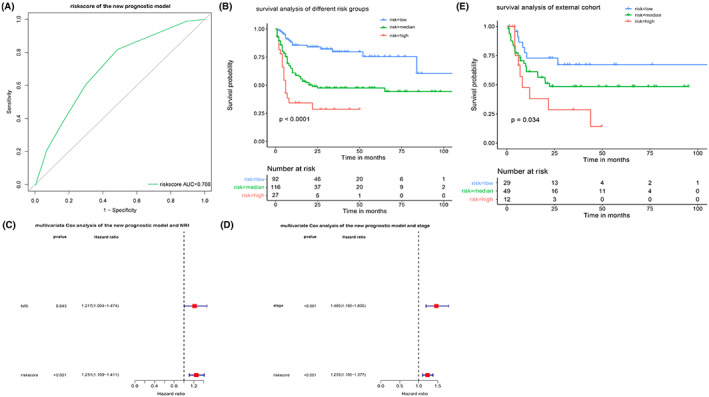
The new prognostic model is meaningful and can be validated by external cohorts. (A) ROC curve of risk score. (B) Survival analysis of different risk groups (low, 0–2; medium, 3–5; high, ≥6) (*p* < 0.0001). (C, D): Multivariate Cox analysis of the new model and NRI or stage. (E) Survival analysis of different risk groups in an external validation cohort (low, 0–2; medium, 3–5; high, ≥6) (*p* = 0.034). ROC, receiver operating characteristic curve.

Finally, we scored each patient using the new model and plotted a new ROC curve (Figure 4A). To render the new prognostic model more convenient for clinical application and facilitate memorization, we stratified patients into three risk levels (low, medium, and high), and the fitting survival curve was also meaningful (Figure 4B). Simultaneously, our results were verified in a separate external validation cohort of 90 patients (Figure 4E and Figure S3). To verify the independence of the novel risk score, we conducted a multivariate Cox analysis (Figure 4C,D), which revealed that the new prognostic model was independent of the Ann Arbor stages and the NRI model.

### 3.5 The novel NRI‐based prognostic index could better stratify patients with ENKTCL

**FIGURE 5:**
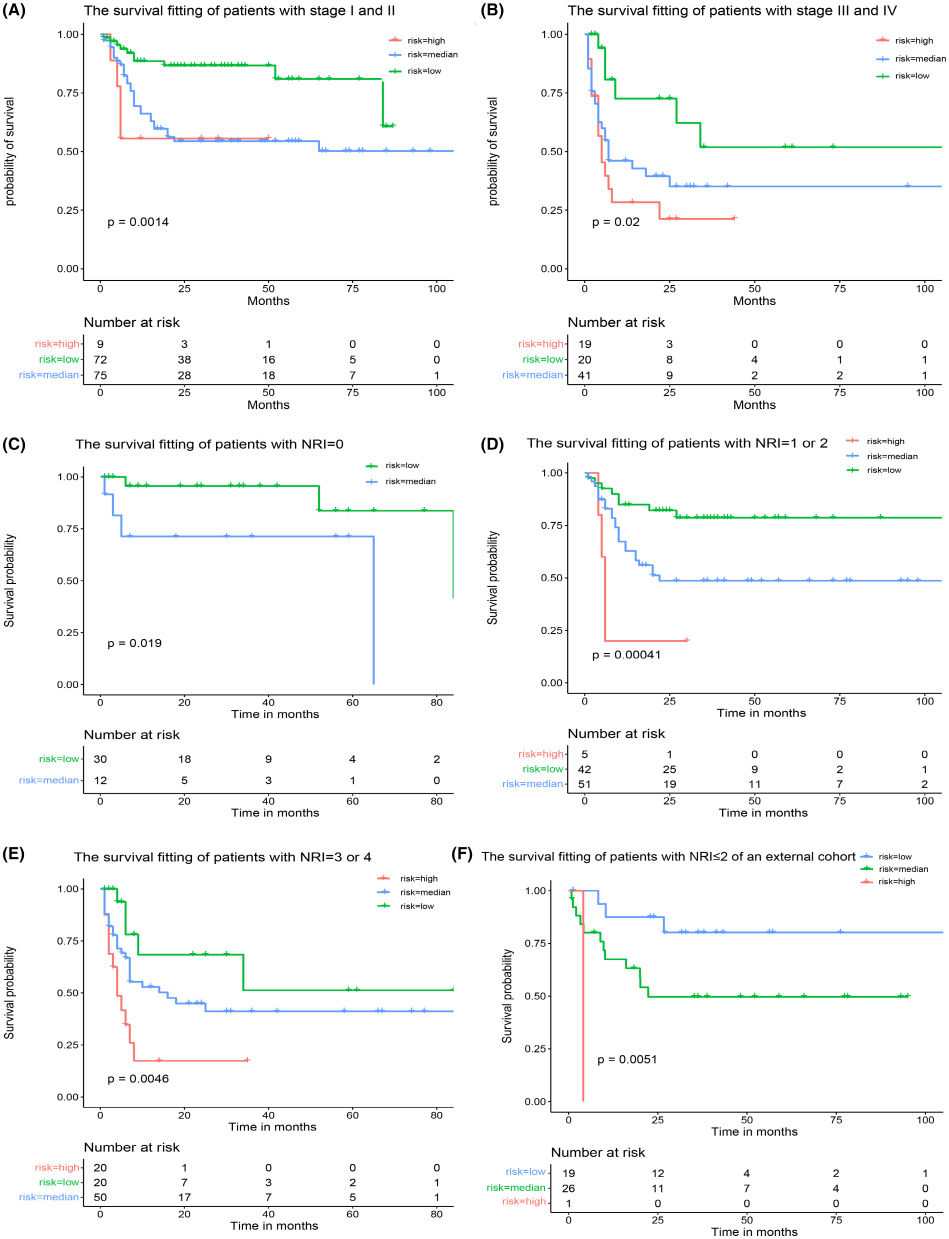
The new prognostic model can further distinguish the risk stratification based on NRI. (A, B) Survival analysis of patients with early‐ and late‐stage disease based on the new prognostic model (*p* = 0.0014 0.02). (C–E) Survival analysis of patients with different NRI based on the new prognostic model (*p* = 0.019, 0.00041, and 0.0046). (F) Survival analysis of patients with NRI = 0–2 based on the new prognostic model in an external validation cohort (*p* = 0.0051). NRI, nomogram‐revised risk index.

As shown in Figure 5A,B, this novel NRI‐based prognostic model could further group the early‐ and advanced‐stage patients (*p* = 0.0014 and 0.02, respectively). Although NRI significantly improved prognostication with respect to the capability for discrimination and the effectiveness of clinical decision‐making, combined with clinical features such as ferritin concentration, we could achieve a more detailed stratification of the prognosis of patients based on NRI. In patients with NRI of zero point, that is low risk, we could effectively further distinguish the risk level using our new prognostic model (Figure 5C). In patients with NRI of 1, that is intermediate low risk, the survival analysis was inaccurate owing to the small number of patients. Therefore, we combined patients with NRI of 1 and 2 to fit the survival data, who were regarded as medium risk of NRI, and found that the model remained applicable (Figure 5D). For patients with high‐risk NRI scores (3 and 4), this novel prognostic model could further stratify patients into different prognostic groups (*p* = 0.0046, Figure 5E). Considering patients with an NRI of 5, survival fitting analysis was not performed, given the limited number of cases. In the external validation cohort, we only performed survival fitting of the new prognostic model for patients with an NRI of 0–2 owing to the relatively small sample size, and the model remained applicable (Figure 5F). Overall, our new prognostic model can further distinguish the risk stratification of patients with ENKTCL based on NRI.

## 4 DISCUSSION

In patients diagnosed with ENKTCL, the current treatment era involves the application of precision medicine or individualized treatment; thus, clinical stratification is particularly important for subsequent treatment selection. As a recently reported index, the prognostic prediction value of NRI has been confirmed. However, we attempted to establish a new prognostic model to subdivide patients with the same NRI and select appropriate treatment regimens with additional individuality. The new model combines the NRI score with multiple clinical indicators.

The serum ferritin concentration is one clinical factor included in this model. Ferritin, an iron‐binding protein,^10^ plays a central role in several metabolic pathways. It is well‐established that elevated serum ferritin can be linked to malignancy and poor outcomes, including potentially devastating diseases such as hemophagocytic syndrome (HLH).^11^ Elevated ferritin levels can reflect an increase in iron reserves throughout the body, but paradoxically, these iron reserves are isolated and unavailable for hematopoietic use, a process that leads to inflammatory anemia.^12^ This relative iron deficiency during inflammation and malignancy is considered a defense mechanism that limits the use of serum iron by pathogens and tumors.^13^, ^14^ Ferritin has been shown to significantly reduce the proliferation and number of granulocyte‐macrophage, erythroid, and multipotential progenitor cells, inducing myelosuppressive responses in patients.^15^ Early in vitro studies have revealed that ferritin regulates immune response by inhibiting lymphocyte function, which depends on interleukin (IL)‐10 production, given that monoclonal antibodies against IL‐10 can attenuate the inhibitory function of ferritin.^16^, ^17^ The mouse T‐cell immunoglobulin‐domain and mucin‐domain 2 (TIM‐2), a member of the T‐cell immunoglobulin and mucin‐domain (TIM) gene family, was the first cell‐surface receptor of ferritin to be cloned. This receptor has a negative regulatory effect on the immune response, potentially indicating that high serum ferritin inhibits immune response.^18^, ^19^ Ferritin may be an attractive target for cancer therapy, given that its downregulation can disrupt the supportive tumor microenvironment, reduce immunosuppression, and enhance sensitivity to chemotherapy. Our analysis also confirmed that ferritin concentration is closely related to the prognosis of ENKTCL.

The hemoglobin concentration is another clinical indicator incorporated into the novel model. Previously, we have examined the relationship between ENKTCL and hemoglobin concentration and found that it can be used to improve the prognostic role of IPI.^20^ Low hemoglobin concentration is known to significantly impact tumor treatment outcomes,^21^ potentially resulting in hypoxia and hypotonia in tissues, thereby impairing immune cell function.^22^ Radiotherapy is critical to mediate a curative effect during early‐stage ENKTCL, and hypoxia can also cause radioresistance, resulting in a poor prognosis in patients with low hemoglobin concentrations.^23^

The third clinical indicator is D‐D. Elevated D‐D levels have been associated with increased tumor burden in solid malignancies, including advanced tumors, regional lymph node involvement, and distant metastases.^24^, ^25^ In hematologic tumors, we have demonstrated that high levels of pretreated D‐D can be associated with severe adverse clinical features and poor survival in NKTCL and is an independent predictor of adverse prognosis.^26^ Therefore, we incorporated D‐D into the new prognostic model.

The albumin concentration is the final factor included in the novel model. The albumin concentration can reflect the nutritional status of the body and has been associated with the prognosis of several cancers.^27^ Studies have shown that malnutrition and inflammation can inhibit albumin synthesis.^28^, ^29^ Furthermore, asparaginase is the main drug used for chemotherapy of ENKTCL, which has been shown to cause liver damage and ultimately reduce albumin production. Thus, low albumin levels in patients with cancer can be attributed to various factors. Although underlying mechanisms remain controversial, the role of serum albumin as a predictor of cancer survival remains indisputable.^30^

We established a new prognostic model by combining the above four clinical features with NRI. Then, to facilitate clinical application, we selected the best cutoff value for the ferritin concentration and the respective scores for all factors. We found that the new prognostic score is generally better than NRI alone. Finally, in patients with the same NRI stratification, we observed that our new prognostic model could further distinguish patients with ENKTCL into low and high risk, despite the limited data. These distinctions are of considerable importance for guiding clinical treatment. Considering patients with low NRI scores and low risk in our new prognostic model, radiotherapy alone could be selected as the treatment choice. In patients with high‐risk factors, commonly used chemotherapy regimens, such as P‐GEMOX, failed to achieve long‐term survival. In recent years, programmed death 1 (PD‐1) inhibitors have shown marked efficacy in relapsed/refractory NK/T‐cell lymphoma,^31^ and a growing number of studies are evaluating the efficacy and safety of first‐line PD‐1 inhibitors combined with chemotherapy.^32^ Moreover, maintenance therapy with PD‐1 inhibitors could be considered in the context of clinical trials for high‐risk patients. Furthermore, as determined in our new prognostic model, anemia or decreased albumin level was considered a risk factor. Thus, more active, supportive care may overcome the negative effects and improve patient tolerance to intensive treatments. However, expanding the sample size by including a large number of qualified patients is essential to further verify our prognostic model. Moreover, our gathered data pertain to China, and additional data need to be included to verify whether our new prognostic model can be applied to Western countries. In addition, we need to further enrich the gene sequencing data of patients to determine the relationship between characteristic genes and factors of the new prognosis model. Subsequently, the new ENKTCL prognostic model can be optimized at the gene level, which would help promote the individualization of treatment.

In summary, our study verified the role of NRI in ENKTCL and combined it with concentrations of ferritin, hemoglobin, D‐D, and albumin to construct a new prognostic model for further stratifying patients with the same NRI risk score. Our new model may improve the effectiveness of clinical treatment decision‐making and provide a new concept for the prognostic evaluation of ENKTCL.

## ETHICS APPROVAL AND CONSENT TO PARTICIPATE

This study was approved by the Ethics Committee of Beijing Tongren Hospital (approval certificate no. TRECKY2020‐022). Given that all personal patient information was de‐identified and anonymized, informed consent from patients was waived.

## AUTHOR CONTRIBUTIONS

**Xindi Liu:** Conceptualization (equal); data curation (equal); formal analysis (equal); methodology (equal); software (equal); validation (equal); writing – original draft (equal); writing – review and editing (equal). **Yuanzheng Liang:** Data curation (equal); formal analysis (equal); methodology (equal); software (equal); validation (equal). **Ziyuan Shen:** Data curation (equal); formal analysis (equal); validation (equal). **Liqiang Wei:** Investigation (supporting); resources (supporting); writing – review and editing (supporting). **Jing Yang:** Data curation (supporting); methodology (supporting); resources (supporting); writing – review and editing (supporting). **Yingshi Piao:** Resources (supporting); writing – review and editing (supporting). **Wei Sang:** Formal analysis (equal); methodology (equal); resources (equal); validation (equal). **Pengfei Li:** Conceptualization (equal); formal analysis (equal); methodology (equal); resources (equal); validation (equal); writing – review and editing (equal). **Liang Wang:** Conceptualization (lead); formal analysis (equal); funding acquisition (lead); project administration (lead); resources (supporting); supervision (lead); writing – original draft (equal); writing – review and editing (equal).

## FUNDING INFORMATION

This work was supported by grants from the National Natural Science Foundation of China (grant nos. 81873450 and 82170181), Beijing Hospitals Authority Youth Programme (code: QML20200201), and Beijing Natural Science Foundation (no. 7222027) to Liang Wang.

## CONFLICT OF INTEREST STATEMENT

The authors declare that they have no competing interests.

## Supporting information


Figure S1:

Figure S2:

Figure S3:

## Data Availability

The data that support the findings of this study are available from the corresponding author upon reasonable request
