# Supplementary figures and images for: A novel prognostic model based on ferritin and nomogram‐revised risk index could better stratify patients with extranodal natural killer/T‐cell lymphoma
Source: Cancer Med. 2023 Mar 16;12(9):10660–71. doi: 10.1002/cam4.5820 (PMC10225229; doi:10.1002/cam4.5820)

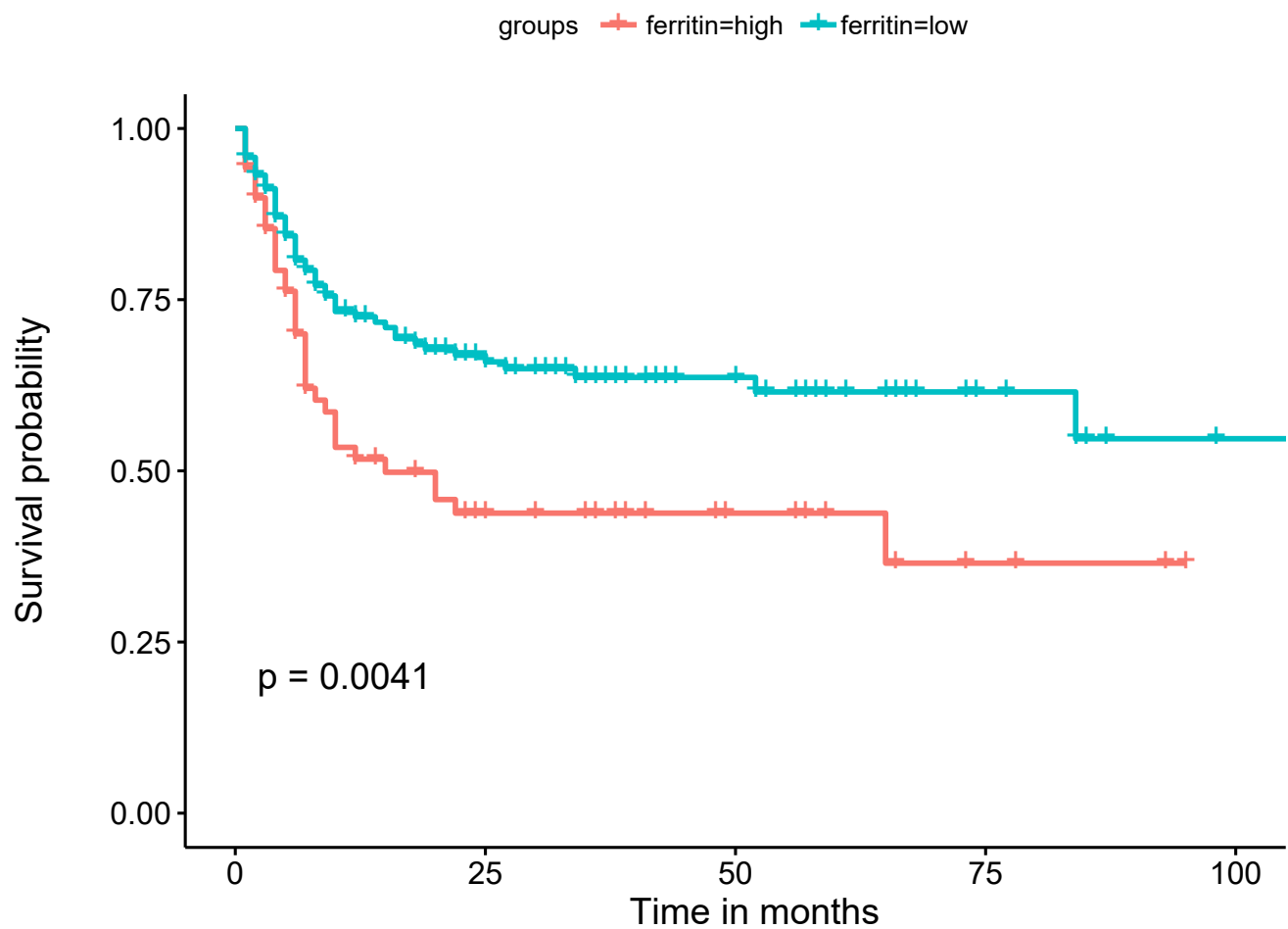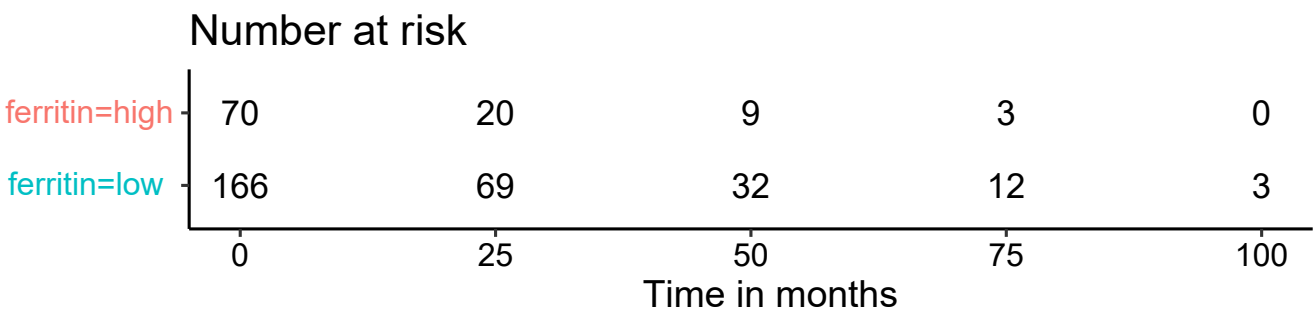

Supplement: Supplementary file 1 — Figure S1: [file CAM4-12-10660-s003.pdf]

Nomogram result of the new prognostic model

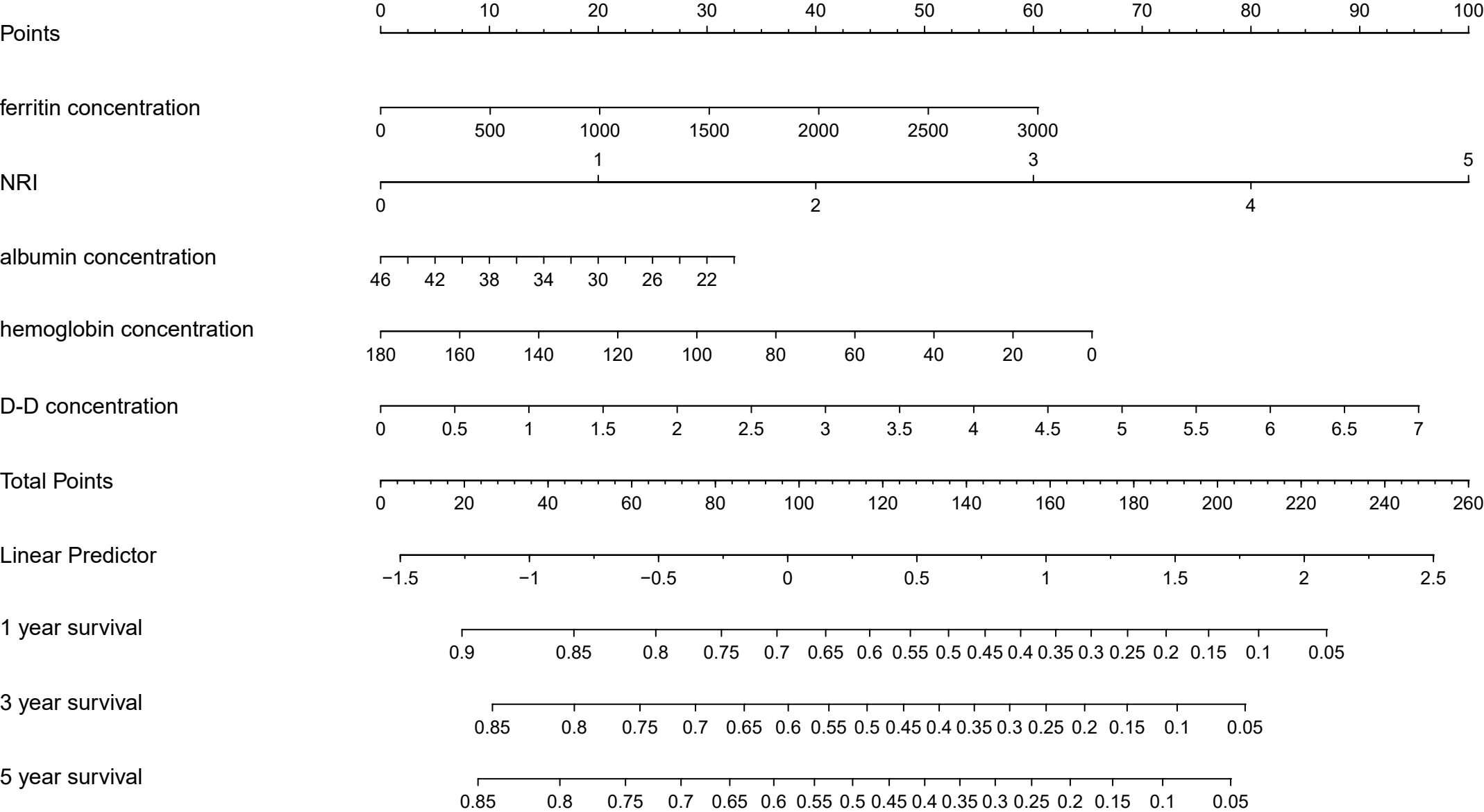

Supplement: Supplementary file 2 — Figure S2: [file CAM4-12-10660-s001.pdf]
