# Supplementary material for: A novel prognostic model based on ferritin and nomogram‐revised risk index could better stratify patients with extranodal natural killer/T‐cell lymphoma
Source: Cancer Med. 2023 Mar 16;12(9):10660–71. doi: 10.1002/cam4.5820 (PMC10225229; doi:10.1002/cam4.5820)
Supplement: Supplementary file 3 — Figure S3: [file CAM4-12-10660-s002.pdf]

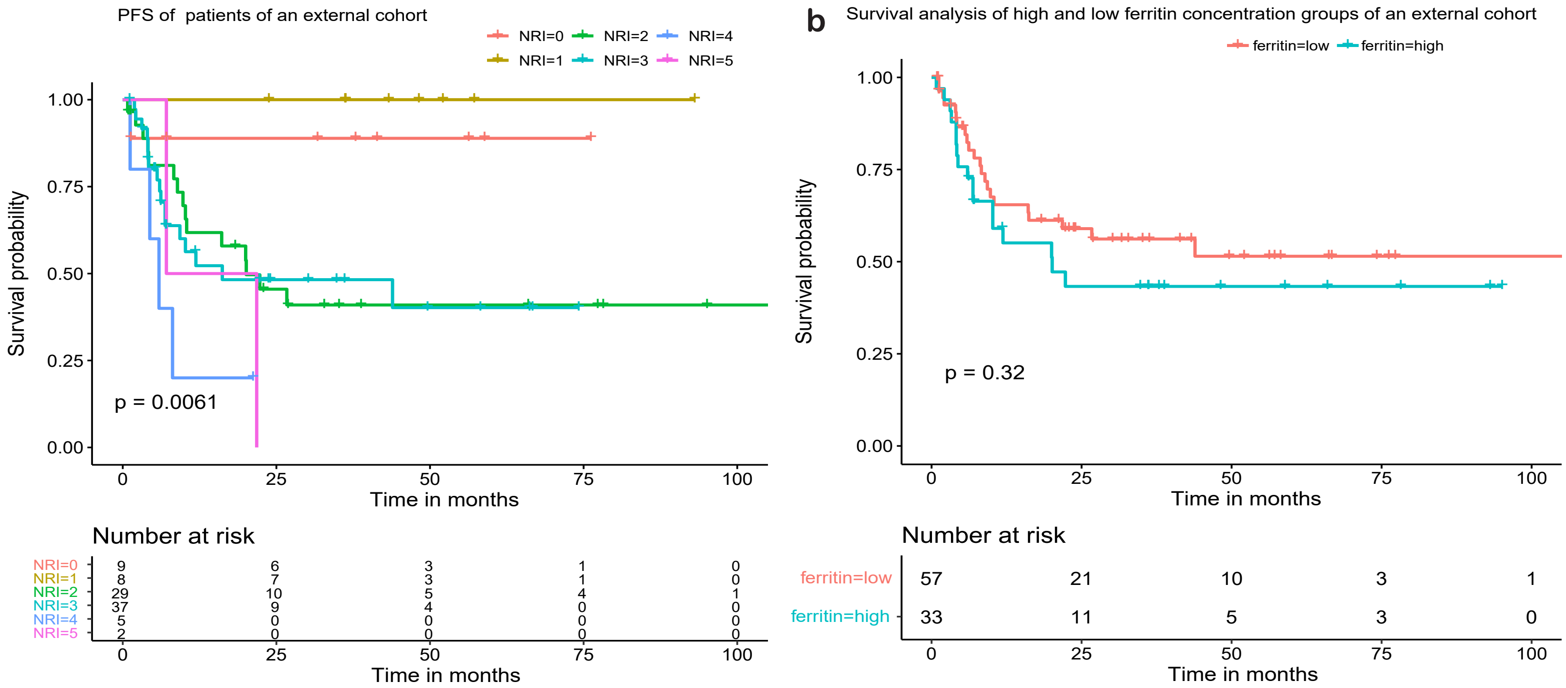

PFS of patients of an external cohort
NRI=0 NRI=2 NRI=4
NRI=1 NRI=3 NRI=5
Survival probability
1.00
0.75
0.50
0.25
0.00
p = 0.0061
0 25 50 75 100
Time in months
Number at risk
NRI=0 9 6 3 1 0
NRI=1 8 7 3 1 0
NRI=2 29 10 5 4 1
NRI=3 37 9 4 0 0
NRI=4 5 0 0 0 0
NRI=5 2 0 0 0 0
0 25 50 75 100
Time in months
b
Survival analysis of high and low ferritin concentration groups of an external cohort
ferritin=low ferritin=high
Survival probability
1.00
0.75
0.50
0.25
0.00
p = 0.32
0 25 50 75 100
Time in months
Number at risk
ferritin=low 57 21 10 3 1
ferritin=high 33 11 5 3 0
0 25 50 75 100
Time in months
